# MicroRNA Let‐7b‐5p Targets IGF1R to Inhibit the Progression of Hepatocellular Carcinoma Through the AKT/mTOR Pathway

**DOI:** 10.1002/cam4.71000

**Published:** 2025-06-18

**Authors:** Jiaojiao Liang, Amin Li, Jun Chen, Niandie Cao, Tao Zhu, Ru Cai, Shuping Zhou, Yong Liang, Xiaolong Tang

**Affiliations:** ^1^ The First Affiliated Hospital of Anhui University of Science & Technology (Huainan First People's Hospital) Huainan China; ^2^ Medical College Anhui University of Science & Technology Huainan China; ^3^ Huainan Maternity and Child Health Hospital Huainan China; ^4^ Central and Clinical Laboratory The Affiliated Huaian Hospital of Xuzhou Medical University and Huaian Second Hospital Huaian China

**Keywords:** AKT/mTOR, hepatocellular carcinoma, insulin‐like growth factor 1 receptor, microRNA let‐7b‐5p

## Abstract

**Background:**

Hepatocellular carcinoma (HCC) remains a major global health burden, with microRNA Let‐7b‐5p (Let‐7b‐5p) emerging as a potential tumor suppressor. However, its precise role and molecular mechanisms in HCC progression remain unclear, necessitating further investigation.

**Methods:**

We assessed Let‐7b‐5p expression in HCC versus normal hepatocytes via qRT‐PCR and employed functional assays (clone formation, EdU, scratch, Transwell, apoptosis) to examine its effects on proliferation, migration, and cell death. Mechanistic studies combined bioinformatics, Western blotting, and IHC to validate IGF1R as a target and assess AKT/mTOR pathway activity.

**Results:**

Let‐7b‐5p was significantly downregulated in HCC cells, and its overexpression suppressed proliferation, migration, and enhanced apoptosis. Mechanistically, Let‐7b‐5p directly targeted IGF1R, leading to reduced phosphorylation of AKT/mTOR, indicating pathway inhibition.

**Conclusions:**

Our findings establish Let‐7b‐5p as a critical regulator of HCC progression via IGF1R‐mediated AKT/mTOR suppression, offering a potential therapeutic strategy for HCC treatment.

## Graphical abstract

Our study highlights the innovative discovery that miRNAlet‐7b‐5p targets the IGF1R and inhibits the AKT/mTOR pathway, which has significant implications in the development of novel therapies to combat HCC, including qRT‐PCR, clone formation assays, EdU experiments, scratch healing experiments, Transwell experiments, PI experiments, and protein immunoblotting.

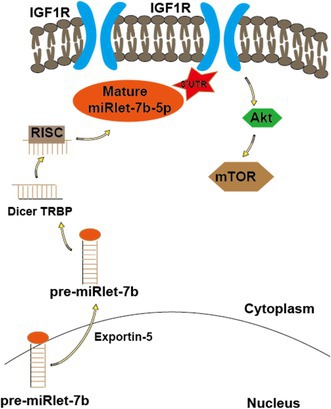

## 1 Introduction

Hepatocellular carcinoma (HCC) ranks as the sixth most common malignancy globally and stands as the third leading cause of cancer‐related mortality [1]. Advanced HCC typically involves first‐line treatments such as chemotherapy, radiation, surgical interventions, and targeted therapies. Despite their prevalence, these approaches often result in substantial toxic side effects, high rates of treatment failure, and the emergence of drug resistance [2, 3]. Consequently, the development of novel, safe, and effective therapeutic strategies for advanced HCC remains a critical imperative.

The etiology of HCC is frequently linked to infections by hepatitis B (HBV) and hepatitis C (HCV) viruses, with HBV being the predominant cause in China [4, 5]. MicroRNAs (miRNAs), which are short, single‐stranded RNA molecules ranging from 19–25 nucleotides, play a significant role in viral pathogenesis and host responses. Originating from pri‐miRNAs through a complex process of recombination and enzymatic cleavage within the nucleus, mature miRNAs are exported to the cytoplasm via exportin‐5 [6, 7]. Here, they contribute to the RNA‐induced silencing complex, targeting the 3′ untranslated regions (3′UTRs) of specific mRNA, leading to either the degradation or inhibition of mRNA translation. This regulation is pivotal in controlling cellular processes such as growth, development, and apoptosis [8]. Interestingly, HCV infection has been shown to upregulate Let‐7b miRNA, which in turn can suppress HCC progression, though the detailed molecular mechanisms remain to be fully elucidated [9, 10, 11]. Recent studies have demonstrated that NEAT1 promotes the proliferation of hepatocellular carcinoma cells and reduces apoptosis by regulating the Let‐7b‐IGF‐1R axis [12]. Let‐7b‐5p inhibits the proliferation and migration of the squamous cell carcinoma cells through negative regulation of KIAA1377 [13]. Additionally, international research has revealed that SNHG16 promotes G2/M phase cell cycle transition by acting on Let‐7b‐5p, thereby advancing liver cancer cell metastasis and epithelial‐mesenchymal transition [14], further underscoring its multifaceted roles in HCC pathogenesis.

The Type I insulin‐like growth factor receptor (IGF1R), a heterotetrameric cell surface tyrosine kinase, plays a crucial role in cellular growth, development, and metabolism [15, 16]. Upon ligand binding, IGF1R activates downstream signaling pathways including the mitogen‐activated protein kinase (MAPK) and phosphatidylinositol 3‐kinase/AKT (PI3K/AKT) pathways, which are integral to cellular proliferation and survival [17, 18]. Our study investigates the potential of Let‐7b‐5p in targeting IGF1R, proposing a novel approach to curb HCC progression through the modulation of the AKT/mTOR signaling axis.

## 2 Materials and Methods

### 2.1 Cell Culture and Reagents

Human Hepatocellular carcinoma cell lines HepG2 and Hep3B, along with the normal liver cell line HHL‐5, were procured from the American Type Culture Collection (ATCC) (Manassas, Virginia, USA). These cell lines were cultured in medium supplied by Gibco medium from Zhejiang Tianxing Biotechnology Co. Ltd., China. The medium was enriched with 10% fetal bovine serum provided by Four Seasons Green Serum (Hangzhou, China). Culturing conditions included maintaining a temperature of 37°C and a CO_2_ concentration of 5%. The culture medium was refreshed every three days. Cell morphology was routinely monitored and assessed. Antibodies targeting IGF1R (#9750), Akt (#4691), phosphorylated Akt (#9916), mTOR (#2983), phosphorylated mTOR (#2974), β‐actin (#4970), and Bcl‐2 (#3498) were sourced from Cell Signal Technology (Danvers, MA, USA). Additionally, the EdU reagent was procured from Beyotime Biotechnology Company (Shanghai, China).

### 2.2 Human Tissue Source

HCC tissues and adjacent non‐tumor tissues (located 2 cm away from the cancerous regions) were procured from 20 HCC patients between January 2021 and January 2023 at the First Affiliated Hospital of Anhui University of Science & Technology. All research involving human participants adhered rigorously to the principles outlined in the Declaration of Helsinki. The study protocol was approved by the Human Research Ethics Committee, and written informed consent was obtained from all participating patients. The clinical characteristics of the enrolled patients are detailed in Table 1.

**TABLE 1:** Clinical and pathological characteristics of liver cancer patients.

Clinicopathological features		Number of cases (*n* = 20)
Sex	Male	14
	Female	6
Age	≤ 50 Years	3
	> 50 Years	17
Number of Tumors	Single	4
	Multiple	16
Tumor Size	≤ 5 cm	4
	> 5 cm	16
Tumor Envelope	Complete	4
	Incomplete	16
TNM Staging	I‐II	1
	III‐IV	19

*Note:* TNM staging refers to the Tumor, Node, Metastasis staging system used for cancer classification.

### 2.3 RT‐qPCR

We used RT‐qPCR to analyze and test the expression levels of Let‐7b‐5p. Total RNA was extracted from Hep3B and HepG2 HCC cells and HHL‐5 cells with Trizol reagent (Shanghai Sangon Biotech #B511311‐0100). The extracted total RNA (3‐4 ng) was reversed as a template for the first‐strand cDNA synthesis by the miRNA stem‐loop RT‐PCR kit (Shanghai Sangon Biotech, China, #B532453‐0020). The qRT‐PCR analysis was performed with the TaKaRa PrimeScript RT Master Mix (Perfect Real Time) kit (Bio‐Link, China #RR820A), and the levels of Let‐7b‐5p expression were detected in Hep3B, HepG2 and HHL‐5 cells. The qPCR conditions were 95°C for 35 min, 60°C for 34 min, and 95°C for 15 min. The amplification was cycled 40 times, and the primersused were Let‐7b‐5p forward F(5′‐AACACGTGTGAGGTAGTAGGTT‐3′) and reverse R(5′‐CAGTGCAGGGTCCGAGGT‐3′); Let‐7b‐5p‐loop (5’‐GTCGTATCCAGTGCAGGGTCCGAGGT ATTCGCACTGGATACGACAACCAC‐3′); U6 stem‐loop primers was used as internal reference: U6 forward F(5′‐AGAGAAGATTAGCATGGCCCCTG‐3′), reverse R(5‐ATCCAGTGCAGGGTCCGAGG‐3′), and U6‐loop (5’‐GTCGTATCCAGTGCAGGGTCCGAGGTATTCGCACTGGATACGACAAAATA‐3′). Relative quantitative expression of Let‐7b‐5p was evaluated using the “Relative expression = 2^−ΔΔCt^” formula.

### 2.4 Adenovirus Transfection

Let‐7b‐5p overexpressing adenovirus and empty adenovirus vector (control) were purchased from Shanghai Sangon Biotech Co. Ltd. (contract number: SGXM2020SIR582‐1). Hep3B and HepG2 cells were seeded into a culture plate and incubated overnight until the cells reached approximately 60% confluence. The Let‐7b‐5p overexpressing adenovirus and empty adenovirus vectors were transfected into Hep3B and HepG2 cells, with an optimal transfection rate of over 80% and strict adherence to the instructions.

### 2.5 Luciferase Experiments

To validate the targeting relationship between Let‐7b‐5p and the IGF1R mRNA, a plasmid termed psiCHECK2‐IGF1R‐3′ UTR was engineered. This plasmid was designed to incorporate the 3′ untranslated region (3′ UTR) of the IGF1R gene, which is purported to be a binding site for Let‐7b‐5p. The Let‐7b‐5p mimic, at a concentration of 50 nM, and the psiCHECK2‐IGF1R‐3′ UTR plasmid were concurrently transfected into 293′T cells using a reliable transfection reagent. Following a 48‐h incubation period, a dual luciferase reporter assay was conducted to assess the luciferase activity. This assay was executed in strict adherence to the manufacturer's protocol for the luciferase assay kit, obtained from Promega, Madison, Wisconsin. The results were meticulously analyzed to determine the extent of suppression of the IGF1R 3′ UTR‐driven luciferase activity by Let‐7b‐5p, thereby confirming the predicted interaction between Let‐7b‐5p and the IGF1R mRNA.

### 2.6 Immunofluorescence

For immunofluorescence, cells were washed with PBS and fixed in acetone for 20 min. After blocking with H_2_O_2_, primary antibodies were added and incubated overnight at 4°C. Cells were then incubated with fluorescein‐labeled secondary antibodies for 60 min at room temperature. DAPI was used to stain nuclei for 10 min. Cells were mounted and coverslipped before examination under fluorescence or laser confocal microscopy.

### 2.7 Immunohistochemistry Analyses

Immunohistochemistry was conducted to evaluate the expression of Ki67 and IGF1R in tissue samples. Following fixation in formalin, the tissues were embedded in paraffin and serial sections, 3 μm in thickness, were prepared. These sections were dewaxed with xylene after antigen retrieval and subsequently incubated with rabbit polyclonal antibodies against Ki67 and IGF1R for 2 h at room temperature. After washing with PBS, the sections were stained with diaminobenzidine (DAB) for visualization. The stained slides were then examined, and the presence of positive staining was recorded under an optical microscope. The intensity and distribution of the immunostaining were carefully observed and documented, providing insights into the cellular proliferation and signaling dynamics associated with IGF1R expression.

### 2.8 EdU Experiment

Cultured HepG2 and Hep3B cells (1 × 10^5^ cells/well) were maintained in 24‐well plates at 37°C with 5% CO_2_ for 24 h. Subsequently, the medium was replaced with EdU‐594 (Beyotime, #C0078S), and the cells were incubated for an additional two hours. Then, fixation with 4% paraformaldehyde, followed by permeabilization with 0.3% Triton X‐100, was performed. The cells were incubated with a click reaction solution for 30 min, washed with PBS, and the nuclei were stained with Hoechst‐33342. The percentage of nuclei stained with red fluorescence was determined using a fluorescence microscope to evaluate cell proliferation.

### 2.9 Scratch Test

In a 6‐well plate, three parallel lines were drawn on the reverse side using a marker pen, and cells were seeded into the wells. Once the cells had proliferated and flattened, three perpendicular lines were scratched into each well using a 10 μL pipette tip. Supernatant was removed, the wells were washed three times with fresh PBS, and 2 mL of serum‐free medium was added. Cell migration was assessed by observing the scratch closure at 0, 24, and 48 h under a fluorescence microscope.

### 2.10 Transwell Assay

Transwell assays with 80 μm diameter filters (JET BIOFIL, Guangzhou, China) were set up in a 24‐well plate. Cells (3.0 × 10^4^ cells/mL) in 100 μL of serum‐free medium were seeded in the upper chamber, while 600 μL of medium was added to the lower chamber. The plate was incubated at 37°C for 24 h. Non‐migrated cells were removed with sterile swabs, and migrated cells were fixed with 4% paraformaldehyde for 15 min, followed by staining with 0.5% crystal violet for an additional 15 min. Membranes were then washed with PBS, and images were captured under a microscope. This process was repeated at least three times to determine the average number of migrating cells.

### 2.11 Clonogenic Assay

1000 HepG2 or Hep3B cells from different experimental conditions were plated into 6‐well plates. Following a 15‐day culture period, the cells were fixed with 4% paraformaldehyde and then stained with 0.5% crystal violet for 15 min. After washing with PBS, the number of distinct clones in each well was counted. This procedure was replicated at least three times for each experimental group to ensure the reproducibility of the results.

### 2.12 Western Blot Experiment

Cells were harvested with trypsin and lysed in a buffer containing RIPA, protease inhibitors, and 0.05 M EDTA at pH 8.0, then incubated on ice for 1 h. The lysate, combined with loading buffer, underwent heat denaturation at 100°C for 20 min to prepare for electrophoresis. Following electrophoresis, 25 μL of the protein sample was loaded onto an 8% SDS‐PAGE gel. Proteins were transferred to PVDF membranes using a semi‐dry transfer system at 100 mV and 400 mA for 90 min. The membranes were blocked with 5% BSA for 1 h at room temperature, washed with TBST, and incubated with primary and secondary antibodies overnight and for 1 h, respectively. After washing, the membranes were treated with a chemiluminescent substrate, and the signal was captured by an imaging system for analysis and documentation.

### 2.13 PI Staining

To assess apoptosis in untransfected, adenovirus‐transfected, and negative control cells. Cells were seeded at 1 × 10^5^ per well in 24‐well plates, incubated for 24 h, and then stained with propidium iodide for 15 min in the dark. Following PBS washes, nuclei were stained with Hoechst‐33342 for 10–15 min. The percentage of red‐fluorescent nuclei was quantified using a fluorescence microscope, indicative of apoptosis levels.

### 2.14 Animal Experimentation

Male NOD‐SCID mice, aged 4–5 weeks, were acquired from Nanjing Junke Biotechnology Co. Ltd., located in Nanjing, China. The Ethical Review Committee of the Medical College at Anhui University of Science & Technology granted approval for all procedures involving these animals. To initiate the animal transplantation model, the mice were assorted randomly into three groups. Each group received a subcutaneous injection on the right flank with 1.5 × 10^6^ cells in a 100 μL suspension of HepG2 cells. The groups were designated as the untransfected control (Ctrl), the empty adenovirus control (Ctrl‐OE), and the Let‐7b‐5p overexpression (Let‐7b‐5p‐OE). Tumor volumes were measured every seven days using vernier calipers and calculated with the formula: *V* = (*L* × *W* ^2^)/2, where *V* represents volume, *L* is length, and *W* is width. After a four‐week observation period, the mice were euthanized using isoflurane. The Ethics Committee of the Medical College of Anhui University of Science and Technology approved all experimental protocols.

### 2.15 Statistical Analysis

TargetScan Human 7.2 software was used to predict the potential target gene family members that may bind to Let‐7b‐5p. All data are presented as means ± standard deviations (SD). Statistical analyses were conducted using GraphPad Prism version 5. Each dataset underwent analysis either through the T‐test or one‐way Analysis of Variance (ANOVA), depending on the dataset characteristics. Results were considered statistically significant at a *p* value of less than 0.05.

## 3 Results

### 3.1 Let‐7b‐5p Was Downregulated in Cancerous Tissues and in HCC Cell Lines

**FIGURE 1:**
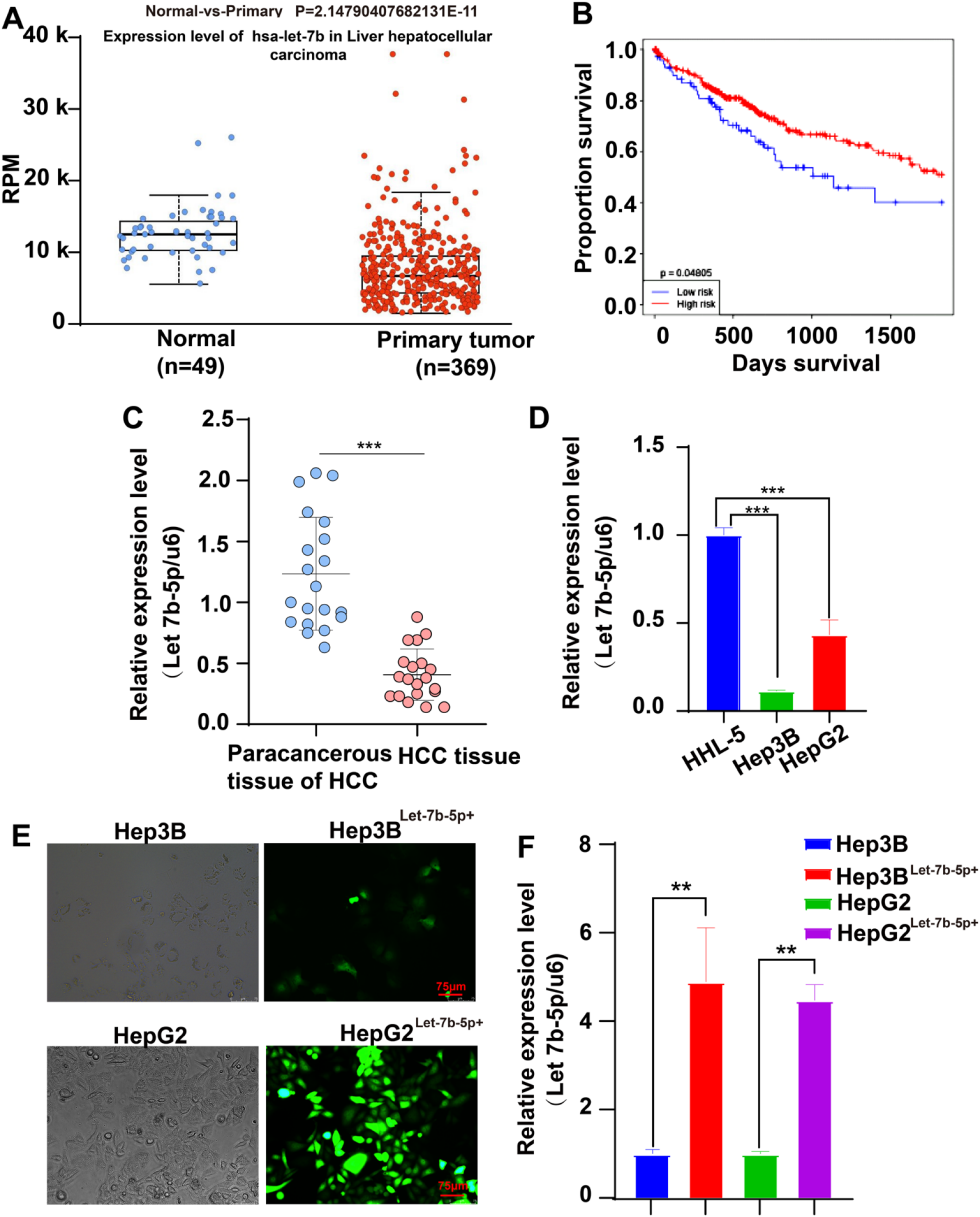
Downregulation of Let‐7b‐5p in cancerous tissues and HCC cell lines. (A) Let‐7b‐5p expression is significantly decreased in cancerous tissues versus normal tissues. This data was derived using the UALCAN database (*p* = 2.15E‐11). (B) Kaplan–Meier curve analysis of Let‐7b‐5p expression and survival rate in HCC patients. (C) Let‐7b‐5p is highly downregulated in HCC tissue compared to non‐tumorous tissue (*p* < 0.001). (D) Let‐7b‐5p is lower in HCC cell lines (Hep3B, HepG2) than in normal liver cells (HHL‐5) (*p* < 0.001). Data were collected from three independent experiments, presented as mean ± standard deviation (*n* = 3). (E) Adenoviral vector transfection efficiency for Let‐7b‐5p overexpression in HCC cells was evaluated. (F) Let‐7b‐5p overexpression was confirmed by qRT‐PCR in HCC cells (*p* < 0.01). **p < 0.01, ***p < 0.001 vs. control group.

Let‐7b‐5p was consistently found to be downregulated in cancerous tissues compared to normal controls, based on data from a comparison between 369 cancerous and 49 normal tissues accessed through the UALCAN database (https://ualcan.path.uab.edu/). This miRNAlet, particularly the 5′ end segment known as Let‐7b‐5p, exhibits a decrease during the maturation process of miRNAlet‐7b, indirectly reflecting the reduced expression in these samples, as illustrated in Figure 1A. The impact of Let‐7b‐5p expression levels on the prognosis of hepatocellular carcinoma patients was assessed using Kaplan–Meier (K‐M) survival analysis. It was found that patients with high Let‐7b‐5p expression exhibited higher survival rates, *p =* 0.04805 (Figure 1B). Additionally, in a study involving HCC patients from the First Affiliated Hospital of Anhui University of Science and Technology, qRT‐PCR analysis of randomly collected cancer tissues showed significantly lower levels of Let‐7b‐5p compared to adjacent non‐cancerous tissues (Figure 1C).

In HCC cell lines (Hep3B and HepG2) and normal liver cells (HHL‐5), Let‐7b‐5p expression was quantitatively analyzed via qRT‐PCR. The results indicated a substantial downregulation in HCC cells compared to HHL‐5 (Figure 1D). Subsequently, overexpression experiments were conducted by transfecting an adenovirus encoding Let‐7b‐5p into the Hep3B and HepG2 cells, achieving approximately 80% transfection efficiency after 48 h (Figure 1E). The successful overexpression of Let‐7b‐5p was confirmed through qRT‐PCR (Figure 1F), facilitating further investigation into its biological effects and mechanistic role in liver cancer.

### 3.2 Let‐7 Inhibits Cell Proliferation at Migration, Promotes Apoptosis

**FIGURE 2:**
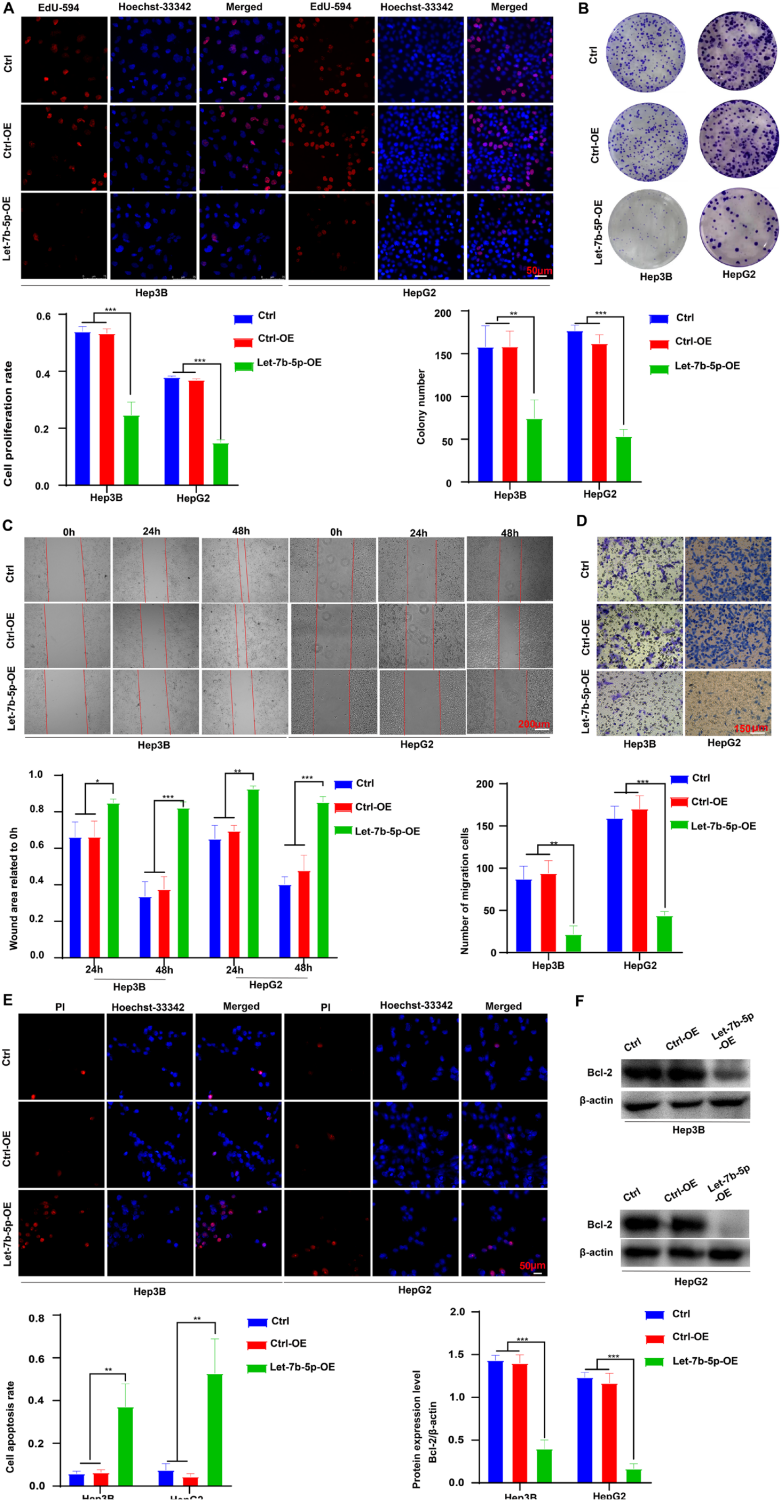
Effects of Let‐7b‐5p on HCC cell functions. (A) Let‐7b‐5p overexpression significantly reduces Hep3B and HepG2 cell proliferation (Let‐7b‐5p‐OE vs. Ctrl‐OE and Ctrl, ***p < 0.001), magnification ×200. (B) Let‐7b‐5p overexpression markedly decreases colony formation in Hep3B and HepG2 cells (Let‐7b‐5p‐OE vs. Ctrl‐OE and Ctrl, **p < 0.01, ***p < 0.001). (C) Cell migration is substantially inhibited in the Let‐7b‐5p‐OE group (vs. Ctrl‐OE and Ctrl, *p < 0.05, **p < 0.01, ***p < 0.001), ×100. (D) Transwell assays confirm a similar suppression of migration (Let‐7b‐5p‐OE vs. Ctrl‐OE and Ctrl, **p < 0.01, ***p < 0.001), magnification ×200, scale bar = 75 μm. (E) Apoptosis is significantly elevated in the Let‐7b‐5p‐OE group (vs. controls, **p < 0.01), magnification ×200. (F) Let‐7b‐5p overexpression significantly lowers Bcl‐2 levels compared to controls (***p < 0.001). Data represent means from three independent experiments (±SD), *n* = 3.

To explore the impact of Let‐7b‐5p on hepatocellular carcinoma (HCC) cell dynamics, we assessed the proliferation of Hep3B and HepG2 cells with overexpressed Let‐7b‐5p through EdU incorporation and colony formation assays. The results, illustrated in Figure 2A,B, reveal a marked reduction in cell proliferation in the Let‐7b‐5p overexpression (Let‐7b‐5p‐OE) group compared to both the empty adenovirus control (Ctrl‐OE) and the untransfected control (Ctrl). Further investigations into cell migration revealed that Let‐7b‐5p overexpression markedly decreased the mobility of these cells, as depicted in Figure 2C,D. This reduction was substantial when compared to the same control groups. Apoptotic rates were analyzed using PI staining and Western blotting techniques, showing a notable increase in apoptosis in cells overexpressing Let‐7b‐5p, relative to controls (Figure 2E,F). Collectively, these results suggest that Let‐7b‐5p acts as a potent inhibitor of cell proliferation and migration, while simultaneously promoting apoptosis in HCC cells. This may be attributed to its regulatory effects on target gene expression and modulation of intracellular signaling pathways, asserting its potential as a therapeutic target in HCC treatment strategies.

### 3.3 IGF1R May Be a Downstream Target Gene of Let‐7b‐5p and Is Highly Expressed in HCC Cells

**FIGURE 3:**
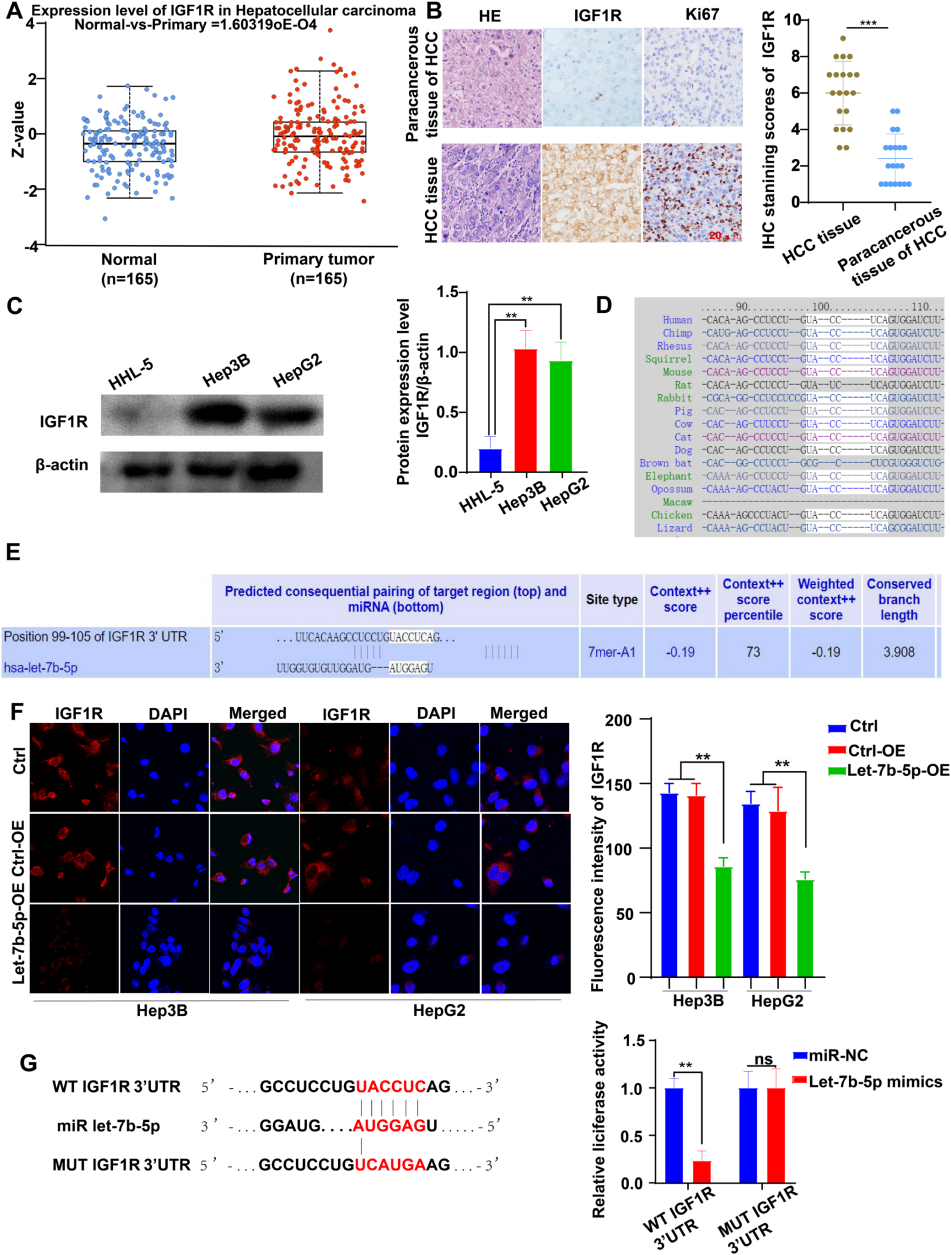
Regulation and expression of IGF1R in HCC and its interaction with Let‐7b‐5p. (A) Analysis using the UALCAN database demonstrates significantly elevated IGF1R expression in HCC tissues compared to normal liver tissues (*p* = 1.603E‐04). (B) Histological assessments, including H&E and immunohistochemical staining, illustrate heightened IGF1R and Ki67 expression in HCC tissues, confirming aggressive cellular proliferation (*p* < 0.001). (C) Western blot analyses reveal increased IGF1R protein levels in Hep3B and HepG2 cell lines compared to the normal liver cell line HHL‐5 (*p* < 0.01). (D, E) Predictive analysis by Targetscan7.2 identifies IGF1R as a potential target of Let‐7b‐5p. (F) Laser confocal microscopy shows reduced IGF1R expression in Hep3B and HepG2 cells following transduction with Let‐7b‐5p adenovirus, highlighting effective miRNA‐mediated downregulation (*p* < 0.01). (G) Luciferase assays demonstrate a significant reduction in luciferase activity in 293 T cells co‐transfected with wild‐type IGF1R 3’‐UTR and Let‐7b‐5p mimics compared to mutants, indicating specific target site interaction (*p* < 0.001 for wild‐type). There was no change in luciferase activity in 293 T cells co‐transfected with the mutated IGF1R vector and Let‐7b‐5p mimics, compared to the controls. Data represent means ± SD from three independent experiments.

After thorough analysis, IGF1R expression was substantially elevated in hepatocellular carcinoma (HCC) tissues compared to normal liver tissues, as demonstrated by the UALCAN database (Figure 3A). Furthermore, histological and immunohistochemical staining revealed a significant upregulation of IGF1R in HCC tissues, particularly in the membranes of cancer cells (Figure 3B). To validate these findings, we conducted Western blot analysis, which revealed that the expression levels of IGF1R were higher in HCC cell lines Hep3B and HepG2 with low Let‐7b‐5p levels, in contrast to the normal liver cell line HHL‐5 (Figure 3C).

To explore the potential regulatory relationship between Let‐7b‐5p and IGF1R, we utilized Targetscan7.2 prediction, which indicated that IGF1R is a target gene of Let‐7b‐5p, and this targeting relationship is evolutionarily conserved (Figure 3D,E). To further investigate the influence of Let‐7b‐5p on IGF1R expression, we performed laser confocal experiments. These experiments demonstrated a significant reduction in the fluorescence intensity of IGF1R expression in Hep3B and HepG2 cells treated with Let‐7b‐5p adenovirus (Figure 3F). Finally, we performed luciferase reporter gene analysis in 293 T cells. The results showed a significant decrease in luciferase activity in 293 T cells co‐transfected with the wild‐type IGF1R vector and Let‐7b‐5p mimic, compared to the control. In contrast, there was no change in luciferase activity in 293 T cells co‐transfected with the mutated IGF1R vector and Let‐7b‐5p mimics, compared to the controls (Figure 3G).

These results collectively suggest a negative correlation between Let‐7b‐5p and IGF1R expression in liver cancer, which implies that IGF1R may be a downstream target gene of Let‐7b‐5p in HCC, and Let‐7b‐5p participates in the regulation of IGF1R expression, thereby affecting the progression of HCC.

### 3.4 Overexpression of Let‐7b‐5p Suppresses the Activation of IGF1R/AKT/mTOR Signaling

**FIGURE 4:**
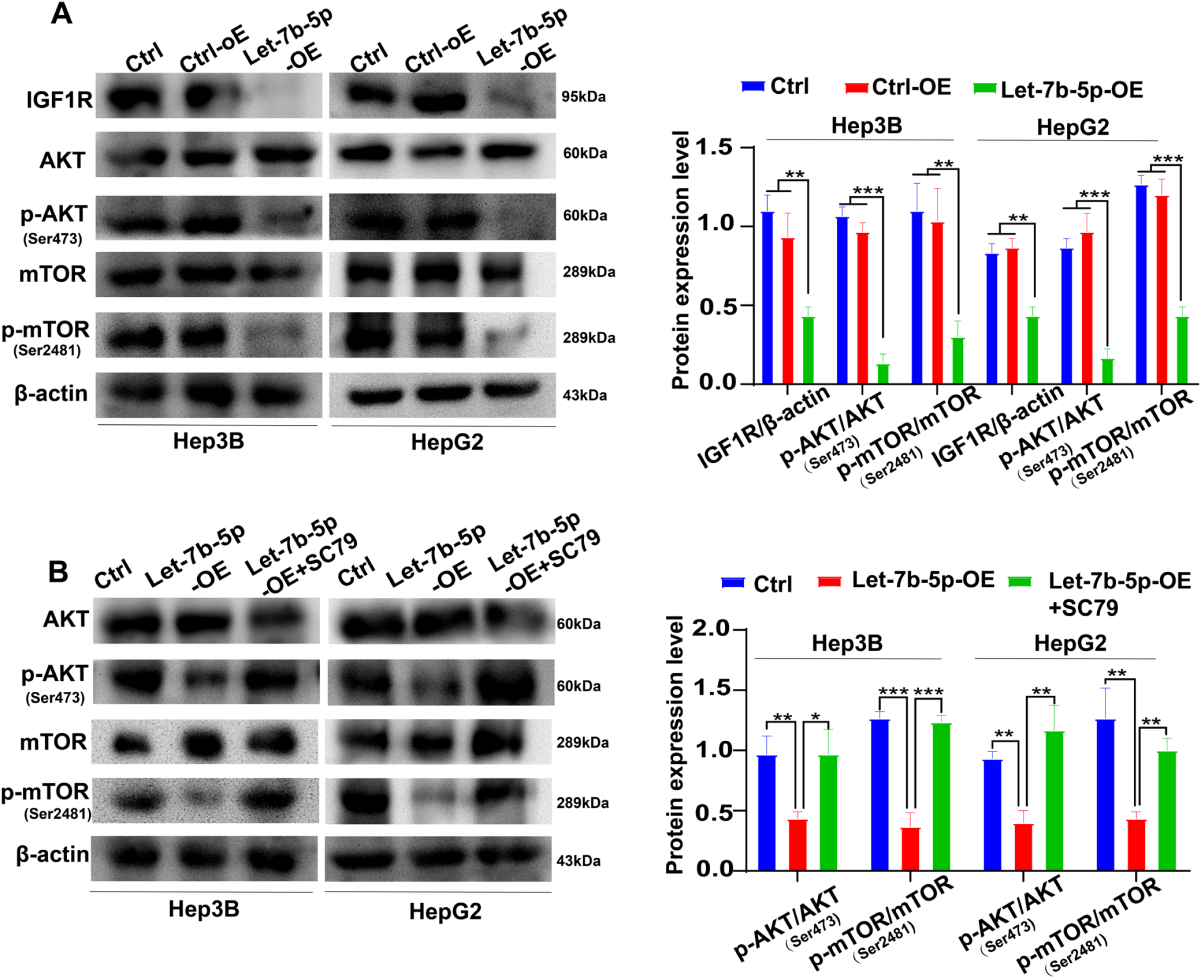
Let‐7b‐5p overexpression inhibits IGF1R/AKT/mTOR signaling activation. (A) Western blot analysis revealed that overexpression of Let‐7b‐5p in Hep3B cells significantly reduced IGF1R expression compared to control groups (**p < 0.01). Concurrently, the activation levels of p‐AKT/AKT and p‐mTOR/mTOR were also attenuated in the Let‐7b‐5p‐OE group (**p < 0.01, ***p < 0.001). Similar effects were observed in HepG2 cells overexpressing Let‐7b‐5p, demonstrating reduced IGF1R expression (**p < 0.01) and dampened p‐AKT/AKT and p‐mTOR/mTOR activation levels (***p < 0.001). (B) Treatment with the AKT phosphorylation activator SC79 increased p‐AKT/AKT and p‐mTOR/mTOR activation levels in Hep3B cells overexpressing Let‐7b‐5p (Let‐7b‐5p‐OE + SC79 group) compared to the Let‐7b‐5p‐OE group (**p < 0.01, ***p < 0.001). This response was also replicated in HepG2 cells overexpressing Let‐7b‐5p following SC79 addition, with elevated p‐AKT/AKT and p‐mTOR/mTOR activation (*p < 0.05, **p < 0.01). Data represent the means ± SD from three independent experiments (*n* = 3).

To investigate the modulatory effects of Let‐7b‐5p on the IGF1R/AKT/mTOR signaling axis, we executed adenoviral‐mediated transfection experiments in Hep3B and HepG2 cells. The successful establishment of Let‐7b‐5p overexpression was confirmed via Western blotting, which showcased marked downregulations in the protein levels of IGF1R, p‐AKT, and p‐mTOR when compared to the control groups (Ctrl‐OE and Ctrl) (Figure 4A). These findings corroborate that Let‐7b‐5p exerts its inhibitory influence on the AKT/mTOR signaling cascade by dampening the expression of IGF1R, thereby underscoring its potential functional significance in the context of HCC pathobiology. To further confirm the influence of Let‐7b‐5p on the AKT/mTOR signaling pathway, we treated the Let‐7b‐5p overexpressing cells with the AKT phosphorylation activator SC79. The subsequent analysis via Western blotting revealed a significant increase in the ratios of p‐AKT/AKT and p‐mTOR/mTOR in the Let‐7b‐5p‐OE + SC79 group, in contrast to the Let‐7b‐5p‐OE group(Figure 4B). This observation suggests that Let‐7b‐5p effectively suppresses the abnormal activation of the AKT/mTOR signaling pathway by targeting the IGF1R gene. Collectively, these data imply that Let‐7b‐5p may play a crucial role in HCC regulation and provide potential insights for therapeutic interventions.

### 3.5 Activating the AKT Signaling Pathway Reverses the Anti‐Proliferative, Anti‐Migration, and Pro‐Apoptotic Effects of Let‐7b‐5p Overexpression on HCC Cells

**FIGURE 5:**
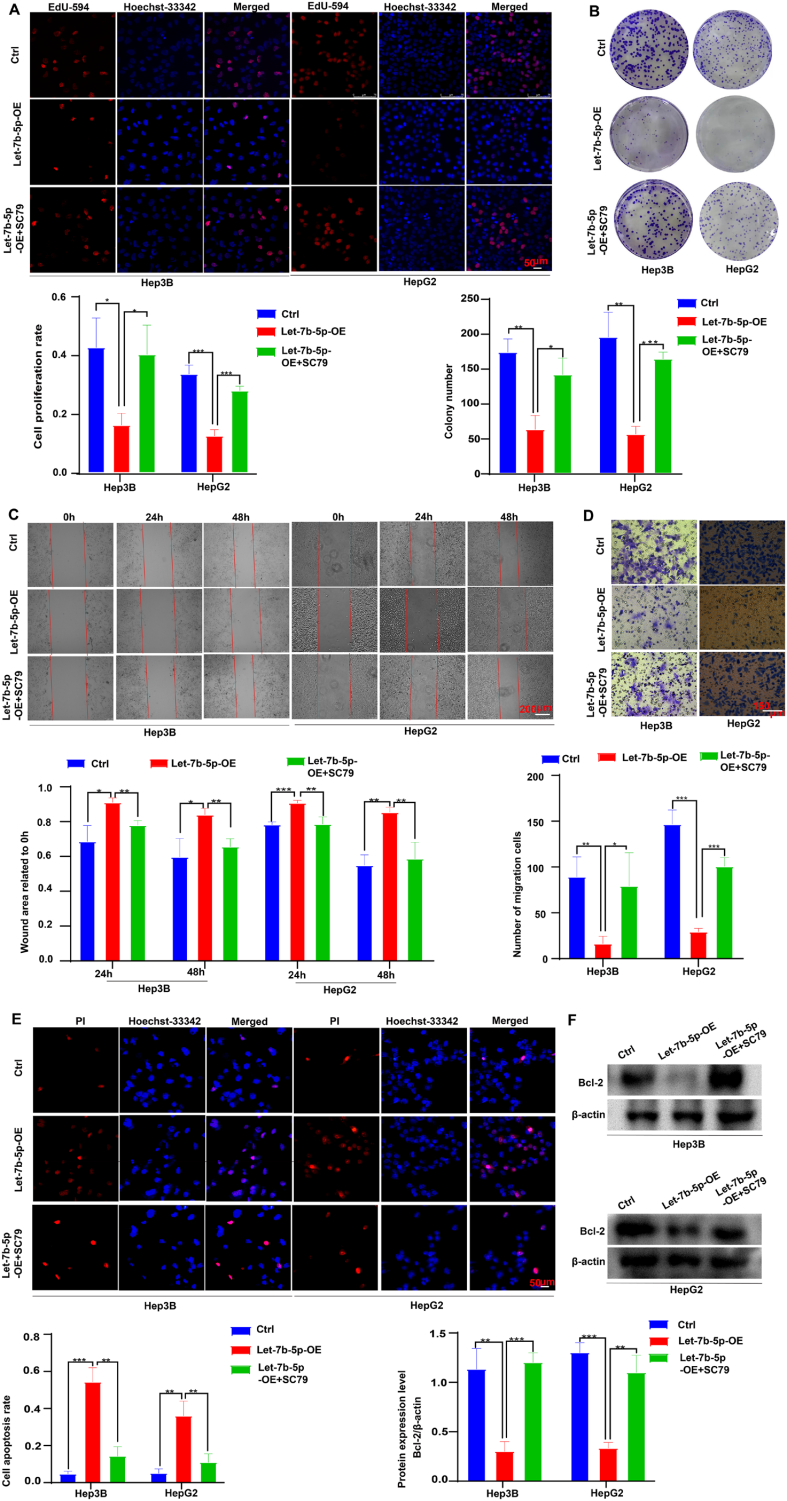
Modulation of Let‐7b‐5p effects on HCC cell function by AKT signaling activation. (A) EdU incorporation assays revealed that Let‐7b‐5p overexpression in Hep3B and HepG2 cells led to increased proliferation, which was further enhanced by the addition of the AKT activator SC79 (Let‐7b‐5p‐OE + SC79 group; *p < 0.05, **p < 0.01, ***p < 0.001 vs. Let‐7b‐5p‐OE group). (B) Colony formation assays demonstrated that the ability of Hep3B and HepG2 cells to form colonies was significantly greater in the presence of both Let‐7b‐5p overexpression and SC79 treatment (Let‐7b‐5p‐OE + SC79 group; *p < 0.05, **p < 0.01, ***p < 0.001 vs. Let‐7b‐5p‐OE group). (C) Scratch assays indicated that the migration of Hep3B and HepG2 cells overexpressing Let‐7b‐5p was potentiated following SC79 treatment (Let‐7b‐5p‐OE + SC79 group; *p < 0.05, **p < 0.01, ***p < 0.001 vs. Let‐7b‐5p‐OE group). (D) Transwell assays revealed that treatment with SC79 enhanced the migratory capacity of Hep3B and HepG2 cells overexpressing Let‐7b‐5p, as compared to the Let‐7b‐5p‐OE group (Let‐7b‐5p‐OE + SC79 group; *p < 0.05, **p < 0.01, ***p < 0.001). (E) Propidium iodide staining demonstrated that the apoptotic rate of Hep3B and HepG2 cells overexpressing Let‐7b‐5p was reduced in the presence of SC79, indicating SC79 rescued the apoptotic effects of Let‐7b‐5p (Let‐7b‐5p‐OE + SC79 group; **p < 0.01, ***p < 0.001 vs. Let‐7b‐5p‐OE group). (F) Western blot analysis showed a significant upregulation of the anti‐apoptotic Bcl‐2 protein in Hep3B and HepG2 cells overexpressing Let‐7b‐5p when treated with SC79 (Let‐7b‐5p‐OE + SC79 group; **p < 0.01, ***p < 0.001 vs. Let‐7b‐5p‐OE group). Data are presented as mean ± SD from three independent experiments (*n* = 3).

To validate the effects of Let‐7b‐5p overexpression in HCC cells, several assays were conducted, including EdU incorporation, colony formation, scratch migration, Transwell migration, PI staining, and Western blotting. The EdU assay and colony formation assays decisively demonstrated that the proliferation of Hep3B and HepG2 cells was significantly enhanced in the presence of both Let‐7b‐5p overexpression and AKT activation (Let‐7b‐5p‐OE + SC79 group), when compared to the Let‐7b‐5p overexpressing group (Let‐7b‐5p‐OE group) (Figure 5A,B). This finding suggests a direct reversal of the anti‐proliferative effects ordained by Let‐7b‐5p upon AKT signaling activation.

Furthermore, the scratch assay and Transwell assay revealed that the migration capacity of Hep3B and HepG2 cells was potently reversed in the Let‐7b‐5p‐OE + SC79 group (Figure 5C,D), indicating an abrogation of the anti‐migration effects typically conferred by Let‐7b‐5p overexpression. The Transwell assay, in particular, yielded a significantly increased number of transmembrane cells, suggesting that AKT activation can indeed counteract the inhibitory influence of Let‐7b‐5p on HCC cell migration.

To elucidate the impact of AKT activation on apoptosis, PI staining and Western blot analysis were conducted. The results unequivocally showed that the pro‐apoptotic propensity of Let‐7b‐5p overexpression was mitigated in the Let‐7b‐5p‐OE + SC79 group, indicating that AKT signaling pathway activation can restore the anti‐apoptotic balance within the cells (Figure 5E). Western blot analysis revealed that the levels of the anti‐apoptotic protein Bcl‐2 were significantly higher in the Let‐7b‐5p‐OE + SC79 group compared to the Let‐7b‐5p‐OE group, further supporting the role of AKT activation in countering the pro‐apoptotic effects of Let‐7b‐5p overexpression (Figure 5F).

In summary, these findings provide robust evidence that the AKT signaling pathway serves as a pivotal regulator that can reverse the inhibitory effects of Let‐7b‐5p on HCC cell proliferation and migration, while also ameliorating the pro‐apoptotic tendency. This regulatory role underscores the critical influence of the AKT/mTOR signaling pathway in the complex network of Let‐7b‐5p's actions on HCC cell biology. The data suggest that modulating AKT activity may represent a viable therapeutic avenue for HCC management by counteracting the deleterious effects associated with Let‐7b‐5p dysregulation.

### 3.6 Inhibition of Let‐7b‐5p Activates the IGF1R/AKT/mTOR Signaling Pathway and Promotes Liver Cancer Progression

**FIGURE 6:**
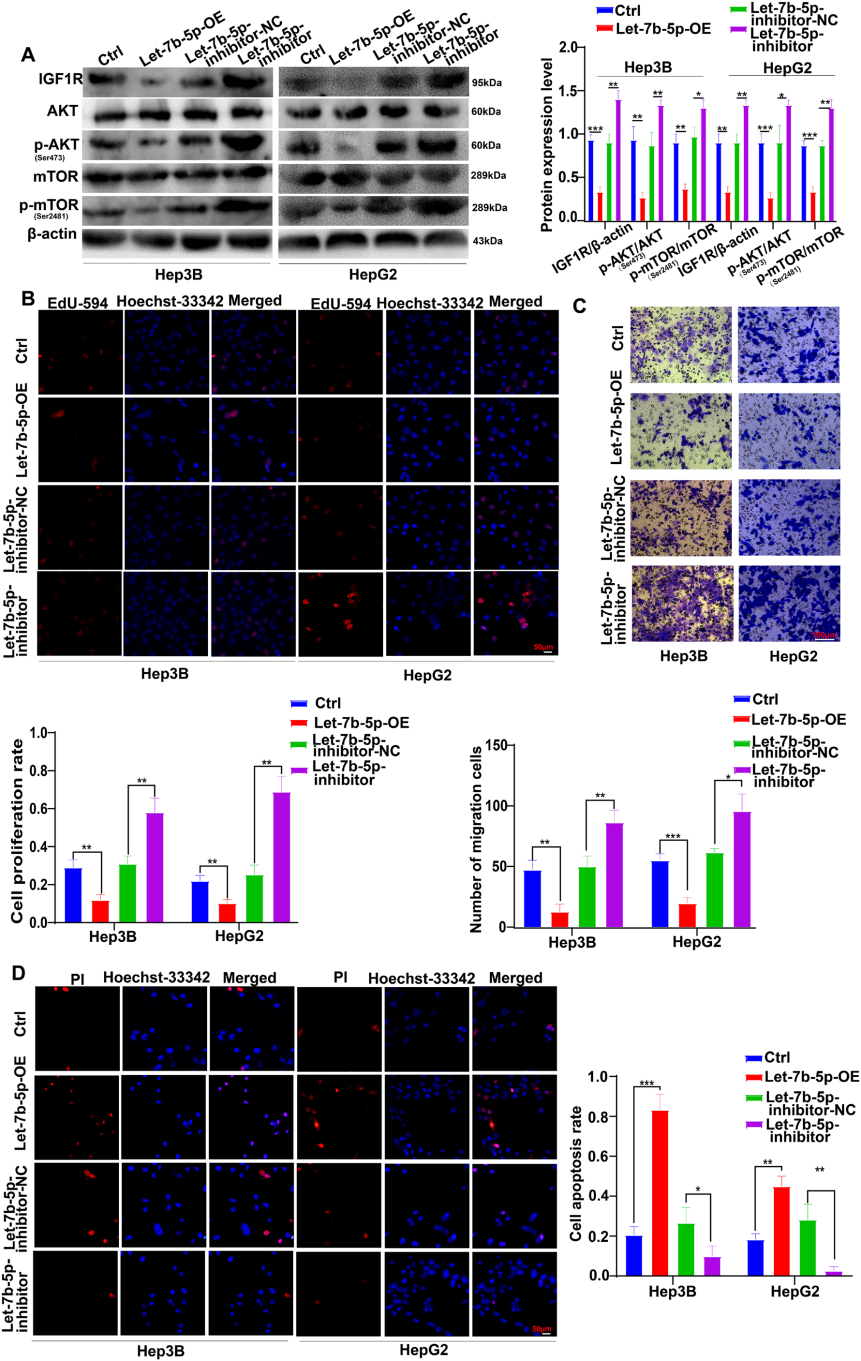
Activation of the IGF1R/AKT/mTOR pathway by inhibition of Let‐7b‐5p promotes liver cancer progression. (A) Western blot analysis shows increased protein levels of IGF1R, p‐AKT, and p‐mTOR in Hep3B and HepG2 cells treated with Let‐7b‐5p inhibitors compared to control and negative control (NC) groups. Significance levels are indicated (*p < 0.05, **p < 0.01, ***p < 0.001). (B) EdU proliferation assays indicate significantly higher cell proliferation in Hep3B and HepG2 cells with Let‐7b‐5p inhibition than in control groups (**p < 0.01). (C) Transwell migration assays demonstrate enhanced migration capabilities in Hep3B and HepG2 cells treated with Let‐7b‐5p inhibitors relative to control groups (*p < 0.05, **p < 0.01). (D) PI staining reveals reduced apoptosis in Hep3B and HepG2 cells with Let‐7b‐5p inhibitors compared to controls (*p < 0.05, **p < 0.01, ***p < 0.001).

To comprehensively validate the interplay between Let‐7b‐5p and the IGF1R/AKT/mTOR signaling pathway in the context of HCC progression, a well‐designed experimental approach was adopted, which included the use of a Let‐7b‐5p inhibitor. Western blot analysis revealed that the inhibition of Let‐7b‐5p resulted in a significant upregulation of the protein levels of IGF1R, p‐AKT, and p‐mTOR in Hep3B and HepG2 cells, as compared to the control groups (Ctrl group, NC group) (Figure 6A).

To elucidate the underlying mechanism further, additional functional assays were conducted. The EdU incorporation assay demonstrated a substantial increase in the proliferation capacity of Hep3B and HepG2 cells in the presence of the Let‐7b‐5p inhibitor, indicating a reversal of the inhibitory effect on cell proliferation (Figure 6B). The Transwell migration assay further corroborated this finding, showing a significant increase in the number of transmembrane cells for both Hep3B and HepG2 cells when the Let‐7b‐5p inhibitor was added, in comparison to the group solely overexpressing Let‐7b‐5p and the control groups (Figure 6C).

PI staining assay provided evidence for a corresponding decrease in apoptosis rates among Hep3B and HepG2 cells in the inhibitor group, suggesting a suppression of the apoptotic effect ordinarily exerted by Let‐7b‐5p (Figure 6D).

Collectively, these findings suggest that the activation of the IGF1R/AKT/mTOR signaling pathway not only reverses the inhibitory influence of Let‐7b‐5p on cell proliferation but also promotes the malignant progression of HCC.

### 3.7 Let‐7b‐5p Inhibited HCC Progression In Vivo

**FIGURE 7:**
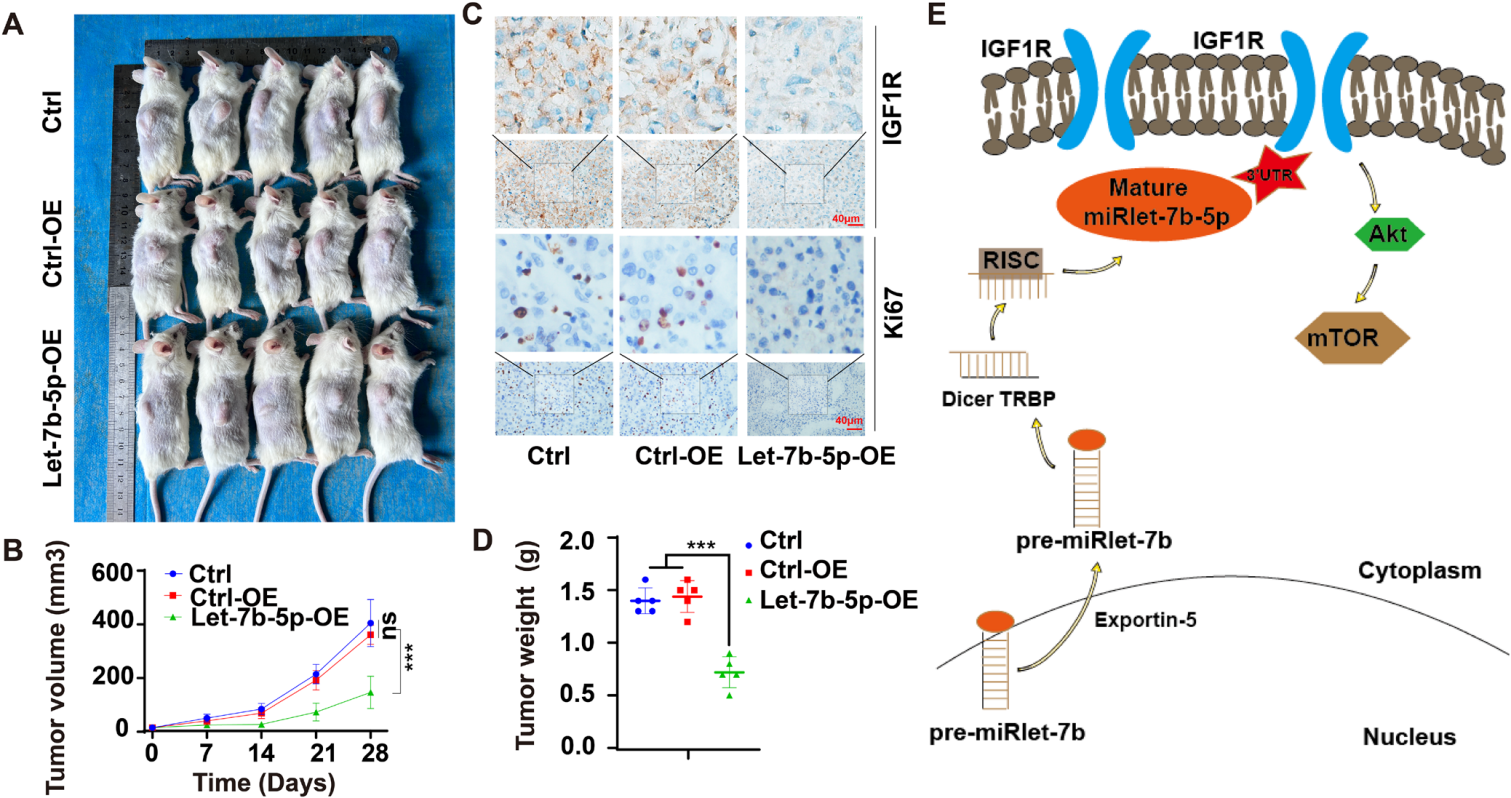
Let‐7b‐5p inhibited HCC progression in vivo. (A) Representative images of mouse xenograft models injected with control cells in each treatment group. (B) Tumor growth rate was significantly curtailed in the Let‐7b‐5p‐OE group compared to Ctrl and Ctrl‐OE groups (***p < 0.001). (C) Quantitative analysis reveals a decrease in the expression of IGF1R and Ki67 in the Let‐7b‐5p‐OE group relative to the Ctrl and Ctrl‐OE groups. (D) Statistical graph of tumor weight outcomes. (E) Let‐7b‐5p inhibits the HCC progression model.

To validate the in vivo inhibitory effect of Let‐7b‐5p on HCC progression, an experimental mouse model was established by injecting HepG2 cells from the Ctrl, Ctrl‐OE, and Let‐7b‐5p‐OE groups into mice. Tumor growth was monitored weekly by measuring tumor sizes and calculating tumor volumes to assess the growth rates. The results revealed that mice in the Let‐7b‐5p‐OE group exhibited significantly smaller tumor sizes compared to the Ctrl and Ctrl‐OE groups (Figure 7A,B). Meanwhile, compared with the Ctrl and Ctrl‐OE groups, the tumor weight of mice in the Let‐7b‐5p‐OE group was significantly smaller (Figure 7D), indicating that Let‐7b‐5p overexpression inhibits HCC growth in vivo.

In addition, to explore the potential mechanism of Let‐7b‐5p in HCC suppression, tumor tissue sections from each group were immunohistochemically stained for the evaluation of IGF1R and Ki67 expression, which are indicative of cell proliferation and signaling pathway activation, respectively. The staining results demonstrated downregulated expression levels of IGF1R and Ki67 in the Let‐7b‐5p‐OE group, suggesting suppressed cell proliferation and signaling activity(Figure 7C), Mechanism diagram of Let‐7b‐5p inhibiting HCC growth (Figure 7E).

Taken together, these in vivo findings provide robust evidence that Let‐7b‐5p overexpression inhibits the growth of HCC tumors, supporting the hypothesis that Let‐7b‐5p plays a crucial role in the retardation of HCC oncogenic progression. This study underscores the potential of targeting Let‐7b‐5p as a therapeutic strategy for the treatment of HCC.

## 4 Discussion

In the present study, we explored the molecular mechanisms underlying the progression of HCC and identified Let‐7b‐5p as a potent suppressor of HCC growth. This microRNA targets IGF1R and dampens the AKT/mTOR signaling cascade. Utilizing qRT‐PCR, we confirmed that Let‐7b‐5p expression was significantly reduced in HCC tissues compared to adjacent non‐tumorous liver tissues, as well as in HCC cell lines when compared to normal liver cells.

As a key member of the miRNAlet‐7 family [19], miRNAlet‐7b has been previously associated with the regulation of hepatitis virus replication and the inhibition of the Wnt/β‐catenin pathway, both of which contribute to the suppression of liver cancer development [20, 21]. Conversely, upregulation of HMGA2 has been linked to the repression of let‐7b expression, potentially facilitating HCC progression [22]. Additionally, the interplay between the PI3K/AKT and JAK2/STAT3 pathways has been suggested to play a role in the inhibition of HCC progression by Let‐7b‐5p [23].

To elucidate the downstream targets of Let‐7b‐5p, we employed the TargetScan7.2 database to predict potential gene targets, including HMGA2, IGF1R, and STAT3, which exhibited high prediction scores. HMGA2, a protein involved in the transcriptional regulation of liver cancer‐related genes, belongs to the high‐mobility group A2 family [24]. STAT3, a critical regulator of cell growth and metabolism, is known to influence the proliferation, migration, and apoptosis of cancer cells [25, 26].

The IGF1R, a receptor tyrosine kinase located on the cell membrane, has been associated with promotional effects on cancer development when overexpressed [27]. In HCC, elevated IGF1R expression has been linked to enhanced cell proliferation, migration, and resistance to apoptosis [27, 28]. Our study demonstrated that Let‐7b‐5p could bind to the 3′ untranslated region (3′UTR) of the IGF1R mRNA, thereby suppressing its expression at both the mRNA and protein levels.

Bioinformatics analysis, Western blotting, immunohistochemical staining, and laser confocal microscopy were employed to investigate the targeting relationship between Let‐7b‐5p and IGF1R (Figures 1 and 3). The protein expression of IGF1R was indeed higher in normal liver cells compared to HCC cells, and the downregulation of Let‐7b‐5p in HCC cells was confirmed (Figures 1, 2, 3). Overexpression of Let‐7b‐5p significantly attenuated IGF1R protein expression in HCC cells, concomitant with a reduction in the fluorescence intensity of IGF1R antibodies in both the cell membrane and cytoplasm (Figures 4, 5, 6).

Furthermore, our findings revealed a negative correlation between the levels of Let‐7b‐5p and IGF1R expression (Figures 4 and 5), suggesting that Let‐7b‐5p can negatively regulate IGF1R‐related biological functions by targeting the IGF1R gene.

Previous research has highlighted the involvement of various intracellular signaling pathways, such as PI3K/AKT/mTOR, MAPK, JAK2/STAT3, and Wnt/β‐catenin, in the occurrence and development of HCC [26, 29, 30, 31]. Activation of the AKT/mTOR signaling pathway has been documented as a critical mechanism in HCC progression, including processes like cell proliferation, epithelial‐mesenchymal transition (EMT), and the regulation of apoptosis and autophagy [32, 33, 34].

Our study demonstrated that the upregulation of Let‐7b‐5p significantly inhibited the phosphorylation of AKT (Ser473) and its downstream mTOR signaling molecules, and decreased the expression of the anti‐apoptotic molecule Bcl‐2 (Figures 4, 5, 6). Using the AKT activator SC79 [35], we confirmed that the AKT/mTOR signaling pathway can counteract the inhibitory effects of Let‐7b‐5p on HCC cell proliferation while promoting apoptosis and inhibiting the migration of liver cancer cells (Figures 5 and 6).

These findings underscore the AKT/mTOR signaling pathway as a pivotal intracellular route governed by Let‐7b‐5p in the regulation of HCC cell behaviors and confirm the role of Let‐7b‐5p in inhibiting HCC progression by regulating cell proliferation, migration, and apoptosis, and identify a potential new treatment target by blocking the AKT/mTOR signaling pathway.

While our study demonstrates the therapeutic potential of Let‐7b‐5p via adenoviral delivery in HCC, it is essential to address potential off‐target effects associated with this approach. Adenoviral vectors, despite their high transfection efficiency, may elicit innate immune responses (e.g., cytokine release) or adaptive immunity against viral capsid proteins, which could limit long‐term therapeutic efficacy [36]. Additionally, miRNAs such as Let‐7b‐5p are known to regulate multiple mRNA targets beyond their primary candidate (IGF1R) due to partial sequence complementarity [37]. For instance, Let‐7 family members have been reported to modulate oncogenes like RAS, HMGA2, and MYC in other cancer contexts [38], raising the possibility of unintended gene regulation in HCC.

To mitigate these risks, our study employed tissue‐specific promoters in adenoviral constructs (though not explicitly stated here) and validated the specificity of Let‐7b‐5p‐IGF1R interaction through luciferase assays and rescue experiments (Figures 3G and 5). Furthermore, in vivo tumor xenograft models showed no significant systemic toxicity (e.g., liver enzyme elevation or weight loss, data not shown), suggesting localized effects. Future studies should incorporate genome‐wide transcriptomic profiling (e.g., RNA‐seq) to comprehensively map Let‐7b‐5p targets and assess bystander effects. Alternative delivery strategies, such as lipid nanoparticles or exosomes engineered for hepatocyte tropism, may further enhance specificity [39].

## 5 Conclusions

Let‐7b‐5p impedes the progression of HCC by targeting the IGF1R, thereby suppressing the AKT/mTOR signaling cascade. These findings suggest a novel therapeutic avenue for HCC, potentially leveraging the modulation of Let‐7b‐5p and the AKT/mTOR pathway to develop innovative treatments.

## Author Contributions

**Jiaojiao Liang:** conceptualization, investigation, methodology, validation, software, formal analysis, data curation, supervision, resources, project administration, visualization, writing – review and editing, funding acquisition, writing – original draft. **Amin Li:** conceptualization, investigation, methodology, validation, software, formal analysis, data curation, funding acquisition. **Jun Chen:** conceptualization, methodology, software, data curation, supervision, formal analysis, validation, investigation. **Niandie Cao:** conceptualization, investigation, funding acquisition, visualization. **Tao Zhu:** conceptualization, methodology, software. **Ru Cai:** conceptualization, methodology, validation, visualization. **Shuping Zhou:** conceptualization, investigation, methodology, validation, formal analysis. **Yong Liang:** conceptualization, methodology, validation, formal analysis, project administration. **Xiaolong Tang:** conceptualization, investigation, funding acquisition, writing – original draft, methodology, validation, visualization, writing – review and editing, software, formal analysis, project administration, data curation, supervision.

## Ethics Statement

This study was approved by the Institutional Review Board of the First Affiliated Hospital of Anhui University of Sciences and Technology, with written informed consent from all patients. The animal study was authorized by the Committee on the Ethics of Animal Experiments of Anhui University of Science and Technology.

## Consent

The authors have nothing to report.

## Conflicts of Interest

The authors declare no conflicts of interest.

## Data Availability

The data and material used to support the findings of this study are included within the manuscript and Supporting Information.

## References

[1] H. Sung , J. Ferlay , R. L. Siegel , et al., “Global Cancer Statistics 2020: GLOBOCAN Estimates of Incidence and Mortality Worldwide for 36 Cancers in 185 Countries,” CA: A Cancer Journal for Clinicians 71 (2021): 209–249. 33538338 10.3322/caac.21660

[2] Y. Wang and B. Deng , “Hepatocellular Carcinoma: Molecular Mechanism, Targeted Therapy, and Biomarkers,” Cancer Metastasis Reviews 42, no. 3 (2023): 629–652. 36729264 10.1007/s10555-023-10084-4

[3] X. Hao , G. Sun , Y. Zhang , et al., “Targeting Immune Cells in the Tumor Microenvironment of HCC: New Opportunities and Challenges,” Frontiers in Cell and Development Biology 9 (2021): 775462. 10.3389/fcell.2021.775462 PMC8633569 34869376

[4] Y. Chen and Z. Tian , “HBV‐Induced Immune Imbalance in the Development of HCC,” Frontiers in Immunology 10 (2019): 2048. 31507621 10.3389/fimmu.2019.02048 PMC6718466

[5] P. Zhao , S. Malik , and S. Xing , “Epigenetic Mechanisms Involved in HCV‐Induced Hepatocellular Carcinoma (HCC),” Frontiers in Oncology 11 (2021): 677926. 34336665 10.3389/fonc.2021.677926 PMC8320331

[6] L. Huang , D. Liang , Y. Zhang , et al., “METTL3 Promotes Colorectal Cancer Metastasis by Promoting the Maturation of Pri‐microRNA‐196b,” Journal of Cancer Research and Clinical Oncology 149, no. 8 (2023): 5095–5108. 36348020 10.1007/s00432-022-04429-9 PMC10349789

[7] M. Billi , E. De Marinis , M. Gentile , C. Nervi , and F. Grignani , “Nuclear miRNAs: Gene Regulation Activities,” International Journal of Molecular Sciences 25, no. 11 (2024): 6066. 38892257 10.3390/ijms25116066 PMC11172810

[8] L. Chen , L. Heikkinen , C. Wang , Y. Yang , H. Sun , and G. Wong , “Trends in the Development of miRNA Bioinformatics Tools,” Briefings in Bioinformatics 20 (2019): 1836–1852. 29982332 10.1093/bib/bby054 PMC7414524

[9] T. L. Liao , Y. M. Chen , S. L. Hsieh , et al., “Hepatitis C Virus‐Induced Exosomal MicroRNAs and Toll‐Like Receptor 7 Polymorphism Regulate B‐Cell Activating Factor,” MBio 12 (2021): e0276421. 34724826 10.1128/mBio.02764-21 PMC8561394

[10] L. Hui , F. Zheng , Y. Bo , et al., “MicroRNA Let‐7b Inhibits Cell Proliferation via Upregulation of p21 in Hepatocellular Carcinoma,” Cell & Bioscience 10 (2020): 83. 32626571 10.1186/s13578-020-00443-x PMC7329548

[11] M. Jamalidoust , M. Shafaati , M. Kalani , M. Zare , and M. Ziyeayan , “MicroRNA Let‐7b Inhibits Hepatitis C Virus and Induces Apoptosis in Human Hepatoma Cells,” Molecular Biology Reports 49, no. 2 (2022): 1273–1280. 34807376 10.1007/s11033-021-06955-0

[12] Q. Liu , H. Shi , J. Yang , and N. Jiang , “Long Non‐Coding RNA NEAT1 Promoted Hepatocellular Carcinoma Cell Proliferation and Reduced Apoptosis Through the Regulation of Let‐7b‐IGF‐1R Axis,” Oncotargets and Therapy 12 (2019): 10401–10413. 31819522 10.2147/OTT.S217763 PMC6890520

[13] S. Zheng , Q. Liu , R. Ma , et al., “Let‐7b‐5p Inhibits Proliferation and Motility in Squamous Cell Carcinoma Cells Through Negative Modulation of KIAA1377,” Cell Biology International 43, no. 6 (2019): 634–641. 30958603 10.1002/cbin.11136

[14] S. Li , F. Peng , Y. Ning , et al., “SNHG16 as the miRNA Let‐7b‐5p Sponge Facilitates the G2/M and Epithelial‐Mesenchymal Transition by Regulating CDC25B and HMGA2 Expression in Hepatocellular Carcinoma,” Journal of Cellular Biochemistry 121, no. 3 (2020): 2543–2558. 31696971 10.1002/jcb.29477

[15] H. Werner , R. Sarfstein , and I. Bruchim , “Investigational IGF1R Inhibitors in Early Stage Clinical Trials for Cancer Therapy,” Expert Opinion on Investigational Drugs 28 (2019): 1101–1112. 31731883 10.1080/13543784.2019.1694660

[16] M. Z. Khan , J. L. Zugaza , and I. Torres Aleman , “The Signaling Landscape of Insulin‐Like Growth Factor 1,” Journal of Biological Chemistry 301, no. 1 (2025): 108047. 39638246 10.1016/j.jbc.2024.108047 PMC11748690

[17] S. S. Zheng , J. F. Wu , W. X. Wu , et al., “CBX1 Is Involved in Hepatocellular Carcinoma Progression and Resistance to Sorafenib and Lenvatinib via IGF‐1R/AKT/SNAIL Signaling Pathway,” Hepatology International 18, no. 5 (2024): 1499–1515. 38769286 10.1007/s12072-024-10696-0 PMC11461582

[18] C. Stefani , D. Miricescu , I. I. Stanescu‐Spinu , et al., “Growth Factors, PI3K/AKT/mTOR and MAPK Signaling Pathways in Colorectal Cancer Pathogenesis: Where Are We Now?,” International Journal of Molecular Sciences 22 (2021): 10260. 34638601 10.3390/ijms221910260 PMC8508474

[19] Y. Ma , N. Shen , M. S. Wicha , and M. Luo , “The Roles of the Let‐7 Family of MicroRNAs in the Regulation of Cancer Stemness,” Cells 10, no. 9 (2021): 2415. 34572067 10.3390/cells10092415 PMC8469079

[20] Y. J. Yeh , C. P. Tseng , S. D. Hsu , et al., “Dual Effects of Let‐7b in the Early Stage of Hepatitis C Virus Infection,” Journal of Virology 95, no. 4 (2021): e01800–e01820. 33208444 10.1128/JVI.01800-20 PMC7851561

[21] Y. Wang , Y. Mo , L. Wang , P. Su , and Y. Xie , “Let‐7b Contributes to Hepatocellular Cancer Progression Through Wnt/β‐Catenin Signaling,” Saudi Journal of Biological Sciences 25 (2018): 953–958. 30108446 10.1016/j.sjbs.2018.03.004 PMC6087813

[22] L. Deng , S. Huang , B. Chen , et al., “Tumor‐Linked Macrophages Promote HCC Development by Mediating the CCAT1/Let‐7b/HMGA2 Signaling Pathway,” Oncotargets and Therapy 13 (2020): 12829–12843. 33363387 10.2147/OTT.S283786 PMC7751845

[23] S. Zhou , Y. Ma , X. Liu , et al., “Targeted Delivery of Glypican 3 (GPC3) Antibody‐Modified MicroRNA (miR Let‐7b‐5p) Polymer Nanoparticles to Sorafenib‐Resistant Hepatocellular Carcinoma Cells,” Journal of Biomedical Nanotechnology 17 (2021): 677–690. 35057893 10.1166/jbn.2021.3033

[24] Q. Zhong , B. Zhao , X. She , and X. Liu , “HMGA2 as a Prognostic and Immune Biomarker in Hepatocellular Carcinoma: Comprehensive Analysis of the HMG Family and Experiments Validation,” PLoS One 19, no. 11 (2024): e0311204. 39591457 10.1371/journal.pone.0311204 PMC11594397

[25] L. Ma , X. Liu , R. Roopashree , et al., “Long Non‐Coding RNAs (lncRNAs) in Cancer Development: New Insight From STAT3 Signaling Pathway to Immune Evasion,” Clinical and Experimental Medicine 25, no. 1 (2025): 53. 39932585 10.1007/s10238-024-01532-8 PMC11813976

[26] X. Zheng , Y. Gou , Z. Jiang , A. Yang , Z. Yang , and S. Qin , “Icaritin‐Induced FAM99A Affects GLUT1‐Mediated Glycolysis *via* Regulating the JAK2/STAT3 Pathway in Hepatocellular Carcinoma,” Frontiers in Oncology 11 (2021): 740557. 34765550 10.3389/fonc.2021.740557 PMC8576446

[27] C. A. Galifi and T. L. Wood , “Insulin‐Like Growth Factor‐1 Receptor Crosstalk With Integrins, Cadherins, and the Tumor Microenvironment: Sticking Points in Understanding IGF1R Function in Cancer,” Endocrine‐Related Cancer 30, no. 10 (2023): e230031. 37490874 10.1530/ERC-23-0031 PMC13418071

[28] M. T. Ngo , H. Y. Jeng , Y. C. Kuo , et al., “The Role of IGF/IGF‐1R Signaling in Hepatocellular Carcinomas: Stemness‐Related Properties and Drug Resistance,” International Journal of Molecular Sciences 22, no. 4 (2021): 1931. 33669204 10.3390/ijms22041931 PMC7919800

[29] D. Guo , D. Zhang , M. Ren , et al., “THBS4 Promotes HCC Progression by Regulating ITGB1 via FAK/PI3K/AKT Pathway,” FASEB Journal 34 (2020): 10668–10681. 32567740 10.1096/fj.202000043R

[30] L. Chen , P. Guo , Y. He , et al., “HCC‐Derived Exosomes Elicit HCC Progression and Recurrence by Epithelial‐Mesenchymal Transition Through MAPK/ERK Signaling Pathway,” Cell Death & Disease 9 (2018): 513. 29725020 10.1038/s41419-018-0534-9 PMC5938707

[31] Q. Li , M. Sun , M. Wang , et al., “Dysregulation of Wnt/β‐Catenin Signaling by Protein Kinases in Hepatocellular Carcinoma and Its Therapeutic Application,” Cancer Science 112 (2021): 1695–1706. 33605517 10.1111/cas.14861 PMC8088956

[32] E. J. Sun , M. Wankell , P. Palamuthusingam , C. McFarlane , and L. Hebbard , “Targeting the PI3K/Akt/mTOR Pathway in Hepatocellular Carcinoma,” Biomedicine 9 (2021): 1639. 10.3390/biomedicines9111639 PMC8615614 34829868

[33] X. Luo , M. Cao , F. Gao , and X. He , “YTHDF1 Promotes Hepatocellular Carcinoma Progression via Activating PI3K/AKT/mTOR Signaling Pathway and Inducing Epithelial‐Mesenchymal Transition,” Experimental Hematology & Oncology 10 (2021): 35. 34088349 10.1186/s40164-021-00227-0 PMC8176587

[34] M. Zhang , S. Liu , M. S. Chua , et al., “SOCS5 Inhibition Induces Autophagy to Impair Metastasis in Hepatocellular Carcinoma Cells via the PI3K/Akt/mTOR Pathway,” Cell Death & Disease 10 (2019): 612. 31406106 10.1038/s41419-019-1856-y PMC6690952

[35] B. Tan , B. Koşar , B. Günaydın Türker , et al., “Akt Activator SC79 Prevents Impaired Subsequent LTP in the Hippocampus of Hypothyroid Rats,” Experimental Brain Research 243, no. 4 (2025): 83. 40032692 10.1007/s00221-025-07025-8

[36] L. Scarsella , E. Ehrke‐Schulz , M. Paulussen , S. C. Thal , A. Ehrhardt , and M. Aydin , “Advances of Recombinant Adenoviral Vectors in Preclinical and Clinical Applications,” Viruses 16, no. 3 (2024): 377. 38543743 10.3390/v16030377 PMC10974029

[37] X. Liu , P. Zhao , X. Du , et al., “Correction: Let‐7b‐5p Promotes Triptolide‐Induced Growth‐Inhibiting Effects in Glioma by Targeting IGF1R,” Naunyn‐Schmiedeberg's Archives of Pharmacology 397, no. 8 (2024): 6271–6272. 38940850 10.1007/s00210-024-03249-7

[38] C. Oliveira‐Mateos , A. Sánchez‐Castillo , M. Soler , et al., “The Transcribed Pseudogene RPSAP52 Enhances the Oncofetal HMGA2‐IGF2BP2‐RAS Axis Through LIN28B‐Dependent and Independent Let‐7 Inhibition,” Nature Communications 10, no. 1 (2019): 3979. 10.1038/s41467-019-11910-6 PMC6726650 31484926

[39] R. Tenchov , J. M. Sasso , X. Wang , W. S. Liaw , C. A. Chen , and Q. A. Zhou , “Exosomes─Nature's Lipid Nanoparticles, a Rising Star in Drug Delivery and Diagnostics,” ACS Nano 16, no. 11 (2022): 17802–17846. 36354238 10.1021/acsnano.2c08774 PMC9706680

